# Isorhamnetin 3-O-neohesperidoside promotes the resorption of crown-covered bone during tooth eruption by osteoclastogenesis

**DOI:** 10.1038/s41598-020-62107-7

**Published:** 2020-03-20

**Authors:** Xijiao Yu, Fuju Zheng, Wenzhi Shang, Yanmei Du, Jinze Zhen, Yi Mao, Shanyong Zhang

**Affiliations:** 1 Department of Oral Surgery, Ninth People’s Hospital, College of Stomatology, Shanghai Jiao Tong University School of Medicine, Shanghai Key Laboratory of Stomatology & Shanghai Research Institute of Stomatology, Shanghai, People’s Republic of China; 2 Department of Endodontics, Jinan Stomatological Hospital, Jinan, Shandong 250001 People’s Republic of China

**Keywords:** Developmental biology, Bone remodelling, Drug regulation

## Abstract

Delayed resorption of crown-covered bone is a critical cause of delayed tooth eruption. Traditional herbal medicines may be good auxiliary treatments to promote the resorption of crown-covered bone. This study was carried out to analyse the effect of isorhamnetin 3-O-neohesperidoside on receptor activator of nuclear factor-kB ligand (RANKL)-induced osteoclastogenesis *in vitro* and resorption of the crown-covered bone of the lower first molars in mice *in vivo*. Isorhamnetin 3-O-neohesperidoside promoted osteoclastogenesis and the bone resorption of mouse bone marrow macrophages (BMMs) and upregulated mRNA expression of the osteoclast-specific genes cathepsin K (CTSK), vacuolar-type H + -ATPase d2(V-ATPase d2), tartrate resistant acid phosphatase (TRAP) and nuclear factor of activated T-cells cytoplasmic 1 (NFATc1). NFATc1, p38 and AKT signalling was obviously activated by isorhamnetin 3-O-neohesperidoside in osteoclastogenesis. Isorhamnetin 3-O-neohesperidoside aggravated resorption of crown-covered bone *in vivo*. In brief, isorhamnetin 3-O-neohesperidoside might be a candidate adjuvant therapy for delayed intraosseous eruption.

## Introduction

The development of osseous eruption is an indispensable stage in the tooth eruption process^1,2^. Osteoclast differentiation is stimulated, causing the resorption of crown-covered bone of an erupting tooth, which forms an intraosseous eruption canal^3^. Osteoclastogenesis in the crown-covered bone is essential. Impaired osseous eruption, in which osteoclastogenesis is disturbed, is common in clinical practice^4^ and can manifest as either delayed or the complete absence of eruption^5,6^. Although unerupted teeth are usually asymptomatic, they may cause cosmetic and pathologic complications^4^ Treatments include orthodontic uprighting, surgical-orthodontic uprighting, surgical uprighting, surgical repositioning, surgical exposure or the removal of pathologic conditions^7^. However, these treatments are very complex and invasive. Research shows that osteoclastogenesis is regulated by a key factor termed receptor activator of NF-κB ligand (RANKL). RANKL agonists or osteoclastogenesis-related drugs can be used to treat delayed tooth eruption^8,9^. Traditional Chinese medicine, which is without toxic side effects may also be a good auxiliary treatment for delayed tooth eruption.

Isorhamnetin 3-O-neohesperidin, known as Pu huang in Chinese^10^, is the main active substance of *T. angustifolia*, can also be isolated from the leaves of *Acacia salicina*^11^. Because of its antioxidant, antiatherogenic and anti-inflammatory activities, Pu Huang has been widely used for the treatment of haematuria, dysmenorrhea, uterine bleeding and trauma in China for a long time^12^. Isorhamnetin 3-O-neohesperidin has been reported to protect cells against oxidative stress by inhibiting H_2_O_2_-induced genotoxicity and DNA damage induced by hydroxyl radicals^13^. Intestinal flora including Escherichia sp. 23 and sp. 30, can convert isorhamnetin 3-O-neohesperidin to three main metabolites, isorhamnetin-3-O-glucoside(I3OG), isorhamnetin and quercetin^14^, which exert various beneficial effects on human health^15^. Isorhamnetin-3-O-glucoside and quercetin were found to exert antioxidant and anti-inflammatory effects on LPS-challenged mouse RAW264.7 macrophage cells^16,17^.

Many traditional antioxidant herbal medicines have been reported to be involved in osteoclastogenesis^18,19^. However, the effect of isorhamnetin 3-O-neohesperidosideon osteoclastogenesis is unclear^20^. In this study, we aimed to determine whether isorhamnetin 3-O-neohesperidoside can regulate the RANKL-induced osteoclastogenesis of bone marrow macrophages (BMMs) *in vitro* and interfere with resorption of crown-covered bone of erupting teeth *in vivo*, to develop new candidate drugs for the treatment of tooth eruption disorders.

## Results

### Cell viability analysis

**Figure 1:**
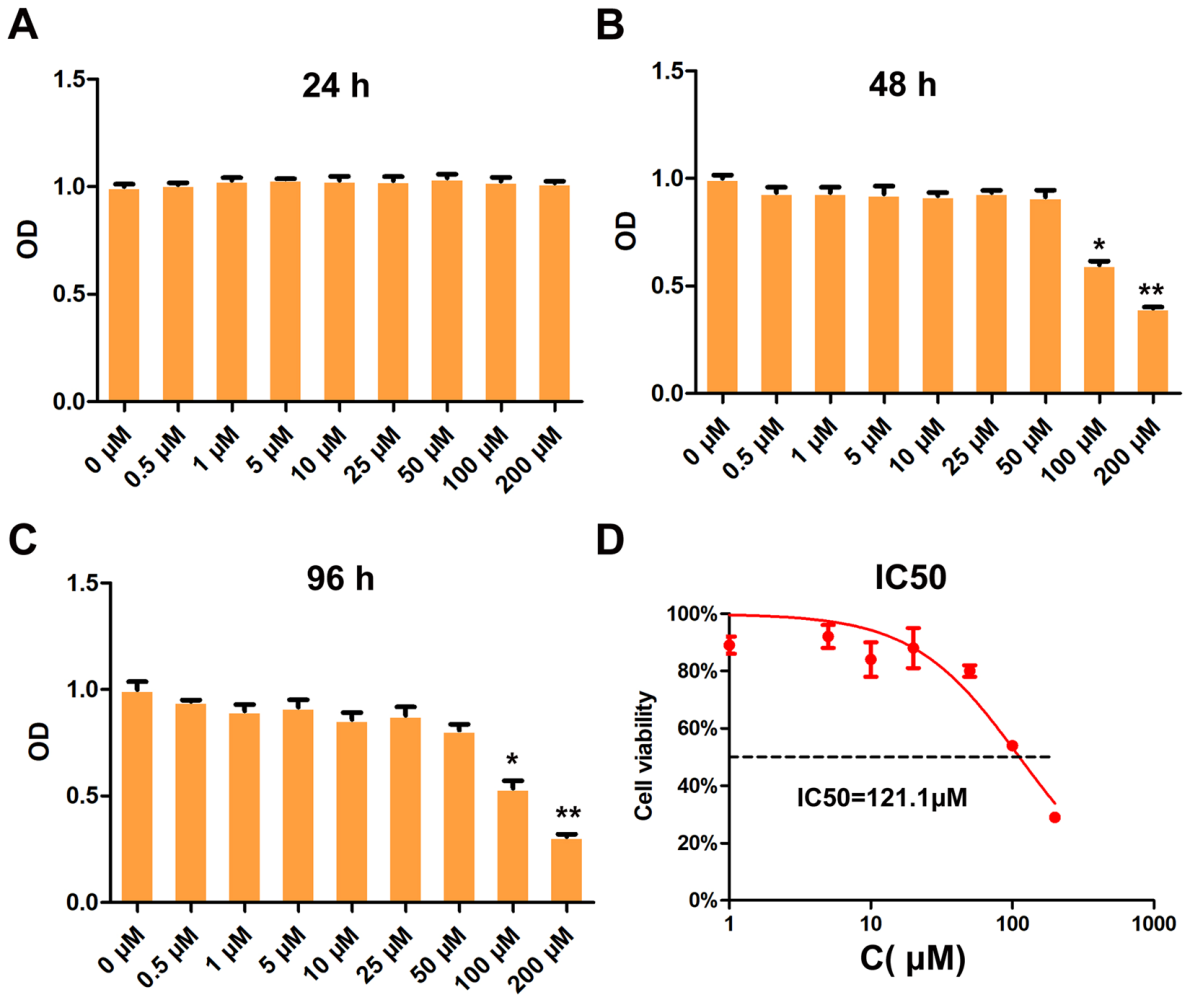
Cell viability determined by CCK‐8 assay. The cell viability of BMMs treated with isorhamnetin 3-O-neohesperidoside (0.5, 1, 5, 10, 25, 50, 100 and 200 μM) for 24 h (**A**), 72 h (**B**) and 96 h (**C**) was detected. (**D**) The half‐maximal inhibitory concentration (IC50) was determined by GraphPad Prism to be 121.1 μM (*p < 0.05, **p < 0.01).

The treatment of BMMs with isorhamnetin 3-O-neohesperidoside at up to 200 μM for 24 h (Fig. 1A) and at up to 50 μM for 48 h (Fig. 1B) and 96 h (Fig. 1C) did not affect cell viability, as shown by CCK‐8 assays. The half‐maximal inhibitory concentration (IC50) of isorhamnetin 3-O-neohesperidoside in BMMs was determined to be 121.1 μM (Fig. 1D). Isorhamnetin 3-O-neohesperidoside at concentrations below 100 μM showed no toxic effects.

### Isorhamnetin 3-O-neohesperidoside promoted RANKL‐induced osteoclastogenesis, as shown by TRAP staining

**Figure 2:**
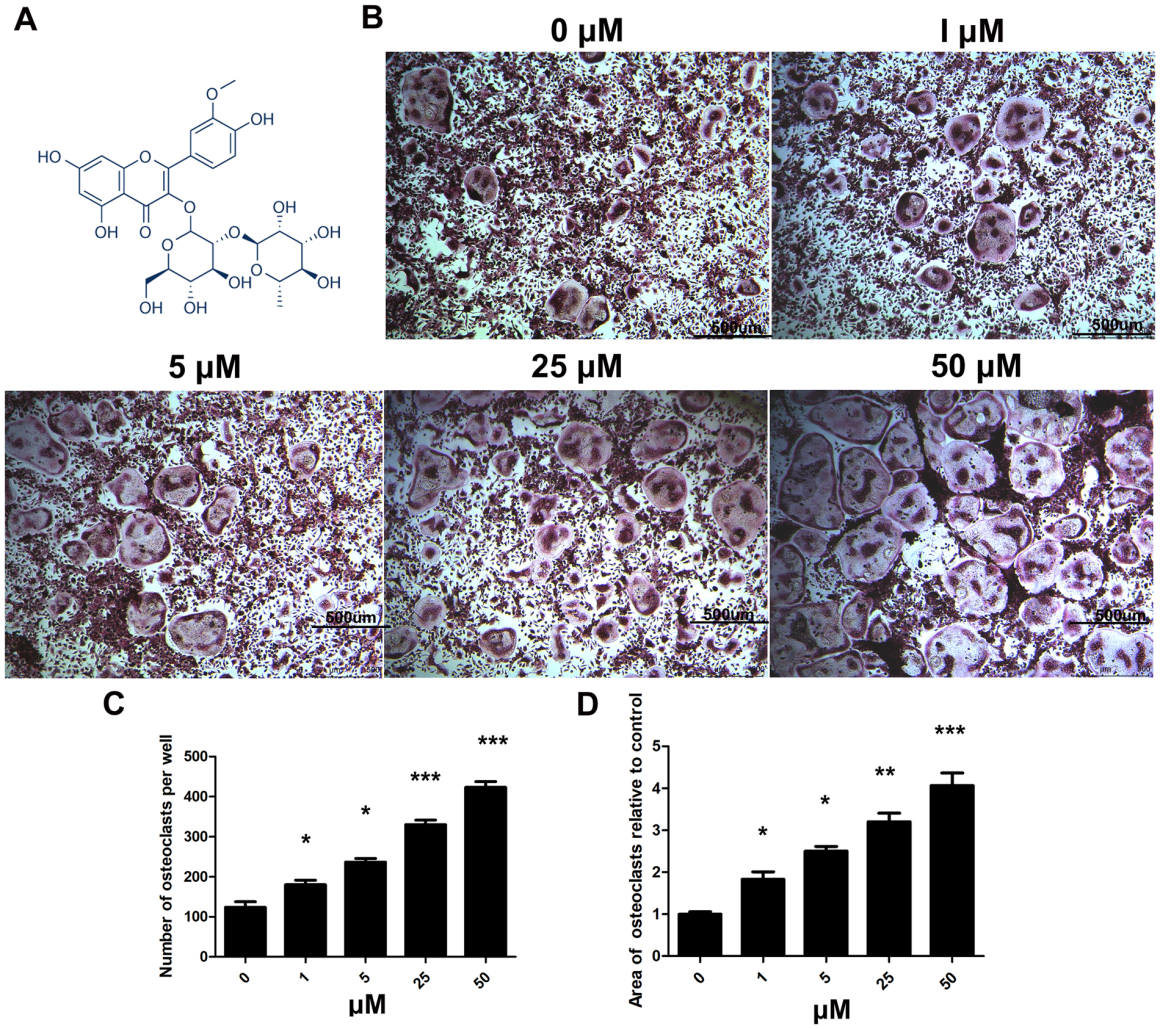
Isorhamnetin 3-O-neohesperidoside promoted RANKL‐induced osteoclastogenesis, as shown by TRAP staining. (**A**) Chemical structure of isorhamnetin 3-O-neohesperidoside. (**B**) BMMs were treated with isorhamnetin 3-O-neohesperidoside (0, 1, 5, 25 and 50 μM) and 50 ng/ml RANKL for 4 days and stained with TRAP. (**C**) Number of TRAP‐positive osteoclasts. (**D**) Area of TRAP‐positive osteoclasts. (*p < 0.05, **p < 0.01, ***p < 0.001).

Chemical structure of isorhamnetin 3-O-neohesperidoside was shown in Fig. 2A. Only a small number of OCs formed after 4 days of induction in the untreated group, but with increasing isorhamnetin 3-O-neohesperidoside concentrations, the number of OCs and OC area increased gradually (Fig. 2B). Isorhamnetin 3-O-neohesperidoside promoted osteoclastogenesis in a dose‐dependent manner (Fig. 2C,D).

### Isorhamnetin 3-O-neohesperidoside promoted bone resorption on Osteo Assay Plates

**Figure 3:**
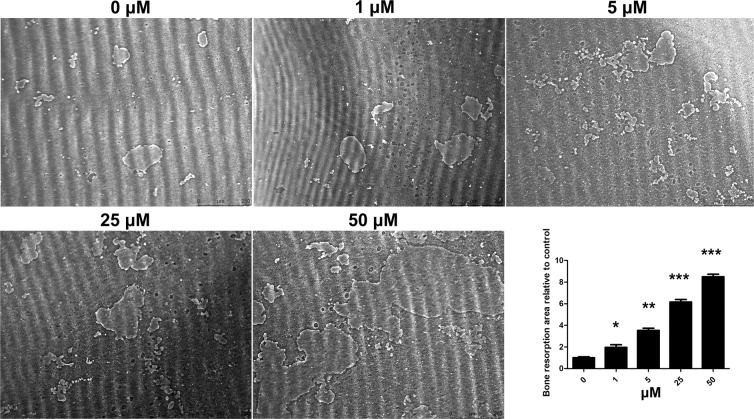
Isorhamnetin 3-O-neohesperidoside promoted osteoclastic bone resorption on 96-well Osteo Assay Plates *in vitro*. The cells were treated with isorhamnetin 3-O-neohesperidoside at the indicated concentrations of (0, 1, 5, 25 and 50 μM) and RANKL (50 ng/ml) for 9 days (*p < 0.05, **p < 0.01, ***p < 0.001).

In the control group, little clearing of the bone biomimetic synthetic surface was observed. However, the resorption area was dose‐dependently increased following treatment with isorhamnetin 3-O-neohesperidoside (Fig. 3).

### Isorhamnetin 3-O-neohesperidoside promoted osteoclast‐specific gene expression

**Figure 4:**
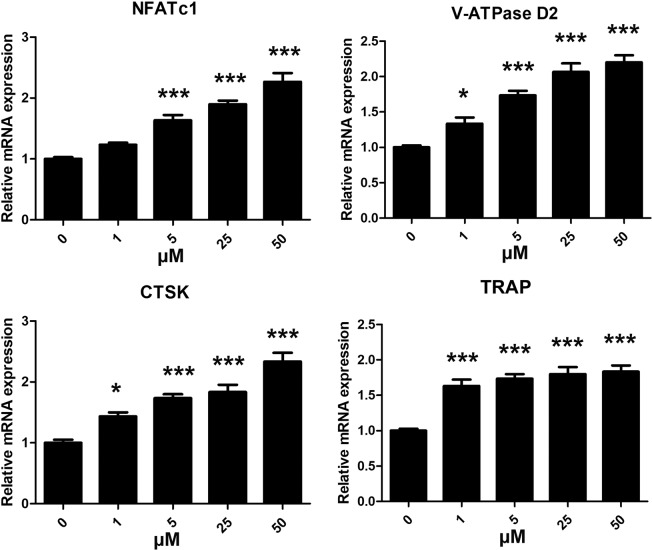
Isorhamnetin 3-O-neohesperidoside promoted osteoclast‐specific gene expression, as shown by real-time PCR. BMMs were treated with isorhamnetin 3-O-neohesperidoside (0, 1, 5, 25 and 50 μM), M‐CSF (30 ng/ml) and RANKL (50 ng/ml) for 4 days. The levels of the osteoclast‐specific genes NFATc1, CTSK, V-ATPase d2 and TRAP were upregulated by isorhamnetin 3-O-neohesperidoside in a dose‐dependent manner (*p < 0.05, **p < 0.01, ***p < 0.001).

Expression of the osteoclast-specific genes NFATc1, CTSK, V-ATPase d2 and TRAP was detected by real-time PCR. Treatment with 1, 5, 25 and 50 μM isorhamnetin 3-O-neohesperidoside significantly upregulated the mRNA levels of NFATc1, CTSK, V-ATPase d2 and TRAP (Fig. 4).

### Isorhamnetin 3-O-neohesperidoside promoted podosome actin ring formation and bovine bone slice resorption

**Figure 5:**
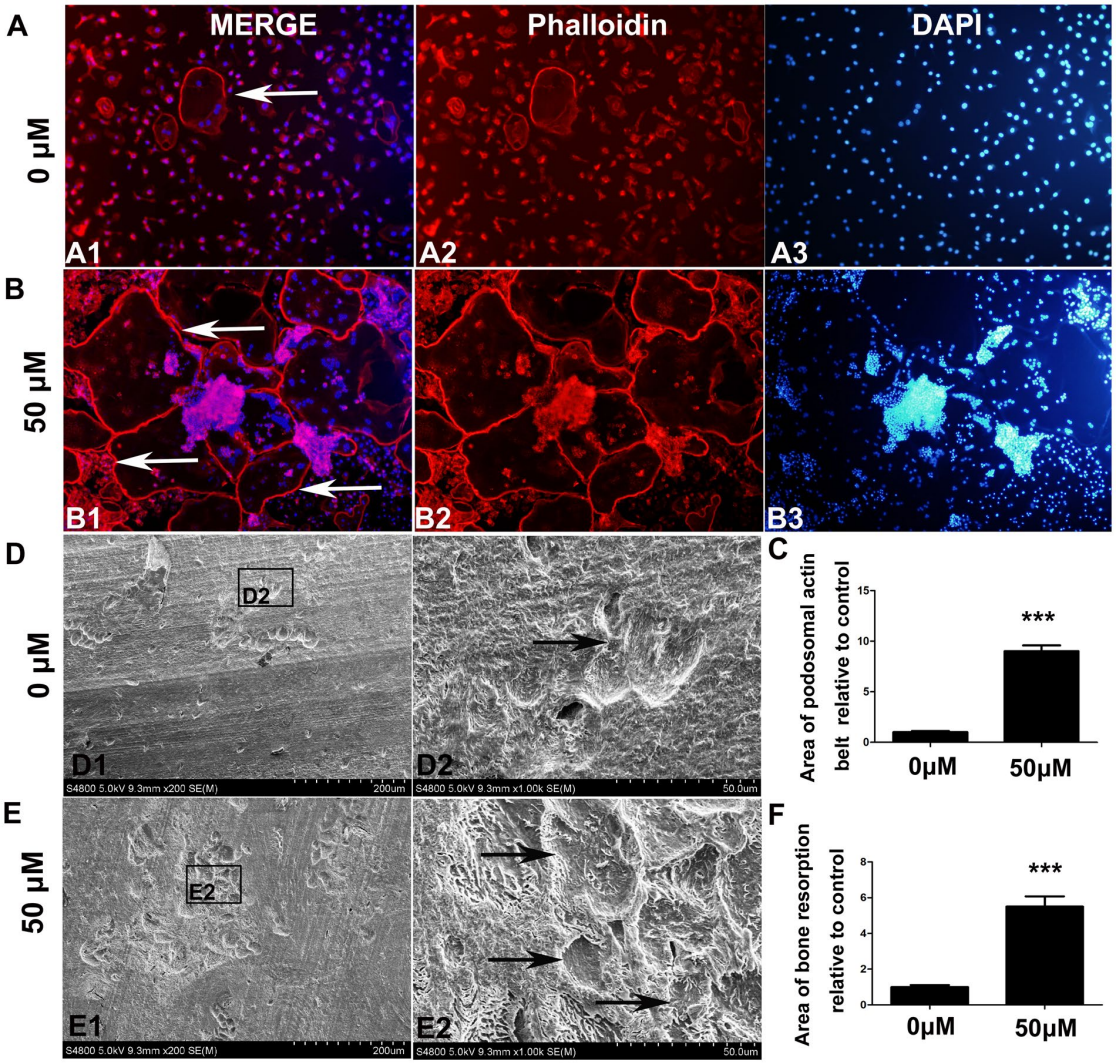
Isorhamnetin 3-O-neohesperidoside promoted bovine bone slice resorption *in vitro*. Immunofluorescence showed the formation of podosomal actin rings (white arrows) in both untreated OCs and 50 μM isorhamnetin 3-O-neohesperidoside-treated OCs (**B**). (**C**)More podosome actin rings were formed in the 50 μM isorhamnetin 3-O-neohesperidoside-treated group. BMMs were treated with isorhamnetin 3-O-neohesperidoside (0 and 50 μM) and RANKL (50 ng/ml) for 9 days. (**D**) Scanning electron microscopy (SEM) showed only a few resorption pits (black arrows) were observed on the bovine bone slice in the untreated group. (**F**) More resorption pits were observed in the 50 μM isorhamnetin 3-O-neohesperidoside-treated group (**E**) than in the untreated group (*p < 0.05, ***p < 0.001).

The results of immunofluorescence analysis showed that 50 μM isorhamnetin 3-O-neohesperidoside promoted podosome actin ring formation in OCs (Fig. 5B), compared with that in the untreated group (Fig. 5A,C). Only a few resorption pits on the bovine bone slices were observed by SEM (Fig. 5D). More resorption pits were observed in the 50 μM isorhamnetin 3-O-neohesperidoside-treated group (Fig. 5E) than in the untreated group (Fig. 5F). Isorhamnetin 3-O-neohesperidoside significantly promoted bovine bone slice resorption.

### Isorhamnetin 3-O-neohesperidoside promoted osteoclastogenesis by upregulating the NFATc1, p38 and AKT pathways

**Figure 6:**
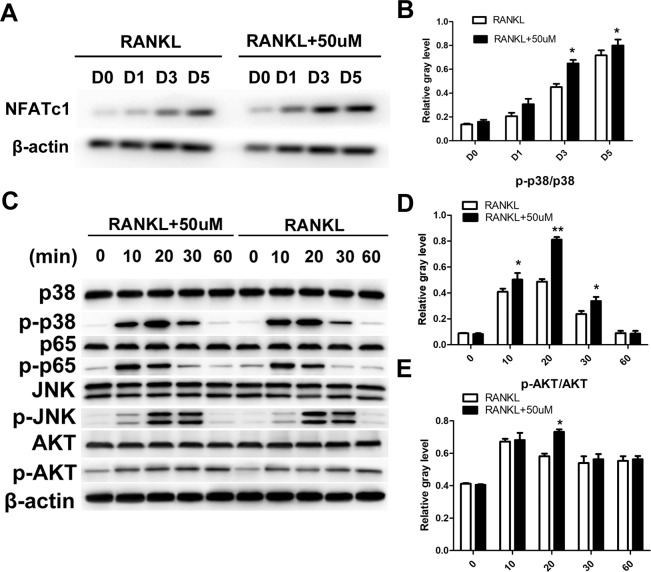
Isorhamnetin 3-O-neohesperidoside upregulated The NFATc1, p38 and AKT pathways, as shown by western blotting. (**A**) BMMs were stimulated by 50 μM isorhamnetin 3-O-neohesperidoside and 50 ng/ml RANKL for 1, 3, and 5 days. (**B**) Isorhamnetin 3-o-neohesperidin promoted NFATc1 expression compared with that in the group treated with only RANKL. (**C**) BMMs were pre-treated with 50 μM isorhamnetin 3-O-neohesperidin for 2 h and then stimulated with 50 ng/ml RANKL for 10, 20, 30 and 60 mins. Total cellular proteins were extracted and analysed. (**D**) The phosphorylation of p38 was significantly enhanced by isorhamnetin 3-O-neohesperidin treatment. (**E**) The phosphorylation of AKT was promoted at 20 min by isorhamnetin 3-o-neohesperidin. (*p < 0.05, **p < 0.01).

The expression of NFATc1 increased gradually from 1 to 5 days after induction with RANKL. NFATc1 expression was increased at days 1, 3 and 5 after isorhamnetin 3-O-neohesperidoside treatment, indicating the positive effect of isorhamnetin 3-O-neohesperidoside on osteoclastogenesis (Fig. 6A,B).

In the group treated with only RANKL, RANKL initiated p38, AKT, p65 and JNK phosphorylation (Fig. 6C, see supplementary files) and the level of p38 phosphorylation was further enhanced by isorhamnetin 3-o-neohesperidin after 10, 20 and 30 min (Fig. 6D). In addition. AKT phosphorylation level at 20 min was promoted by isorhamnetin 3-O-neohesperidin (Fig. 6E).

### Isorhamnetin 3-O-neohesperidoside promoted the resorption of bone crown-covered resorption *in vivo*

**Figure 7:**
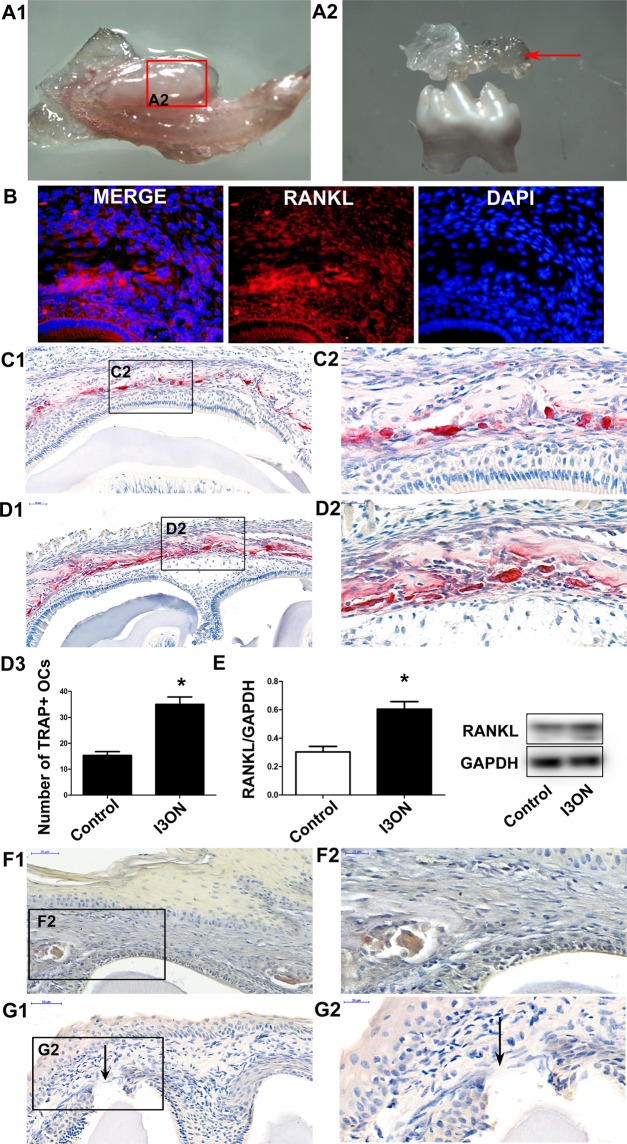
Isorhamnetin 3-O-neohesperidoside upregulated RANKL expression in bone crown-covered bone in the lower first molars of mice *in vivo*. The mouse mandible was separated (**A1**) and fresh crown coverage of dental follicle of the lower first molar was collected for western blotting (red arrow) (**A2**). (**B**) Positive RANKL expression was observed in the crown coverage of dental follicle by immunofluorescence. (**C1**) Many TRAP-positive osteoclasts were observed around the crown-covered bone in the control groups. (**C2**) Higher magnification of black-boxed regions in (**C1**). (**D3**) More TRAP-positive osteoclasts were detected in the isorhamnetin 3-O-neohesperidoside(I3ON)-treated groups (**D1**) than in the control groups at postnatal day 11. (**D2**) Higher magnification of black-boxed regions in D1. (**E**) Western blotting showed that isorhamnetin 3-O-neohesperidoside upregulated RANKL expression in the crown coverage of dental follicle. (**F1**) Unabsorbed crown-covered bone and several TRAP-positive osteoclasts were still observed in the control group at postnatal day 13. (**F2**) Higher magnification of black-boxed regions in F1. (**G1**) Crown-covered bone was completely resorbed and mucosal penetration (black arrow) was initiated in the isorhamnetin 3-O-neohesperidoside-treated groups at postnatal day 13. (**G2**) Higher magnification of black-boxed regions in G1. (*p < 0.05).

After the mouse mandibles were separated, fresh crown coverage was collected from the lower first molars for western blotting analysis (Fig. 7A1,A2). Positive RANKL expression was observed in the crown coverage of dental follicle by immunofluorescence (Fig. 7B). More OCs were observed around the crown-covered bone in the groups treated with isorhamnetin 3-O-neohesperidoside (Fig. 7D1,D2) than in the left mandibular first molar, which served as a control (Fig. 7C1,C2,D3) at postnatal day 11. Western blotting showed that isorhamnetin 3-O-neohesperidoside upregulated RANKL protein expression in the crown coverage (Fig. 7E). Crown-covered bone in the isorhamnetin 3-O-neohesperidoside-treated groups was completely resorbed at postnatal day 13 (Fig. 7G1,G2), but some unabsorbed crown-covered bone and several TRAP-positive osteoclasts were still observed in the control groups (Fig. 7F1,F2). These results showed that isorhamnetin 3-O-neohesperidoside can increase osteoclasts and promote the resorption of crown-covered bone, which is an important stage in tooth eruption.

## Discussion

Until now, there have been no reports discussing the effect of isorhamnetin 3-O-neohesperidoside on osteoclastogenesis. In this study, we found that isorhamnetin 3-O-neohesperidoside increased RANKL-induced osteoclastogenesis in a dose-dependent manner without cytotoxicity. Isorhamnetin 3-O-neohesperidoside strongly promoted osteoclast formation and function *in vitro*. The upregulated levels of the osteoclast-specific genes NFATc1, CTSK, V-ATPase d2, and TRAP shown by qPCR further demonstrated the effect of isorhamnetin 3-O-neohesperidoside in aggravating osteoclastogenesis.

We further investigated the molecular mechanisms by which RANKL-induced osteoclastogenesis is increased by isorhamnetin 3-O-neohesperidoside. NFATc1 is a master transcription factor that regulates osteoclastogenesis^21,22^; NFATc1-deficient osteoclast precursor cells failed to differentiate into osteoclasts in response to RANKL stimulation, while NFATc1 caused precursor cells to undergo efficient osteoclast differentiation without RANKL signalling^21,23,24^. Western blotting showed that the expression of NFATc1 increased gradually from day 1 to day 5 after RANKL induction. However, in the isorhamnetin 3-o-neohesperidin-treated group, the expression of NFATc1 was further increased, indicating that isorhamnetin 3-O-neohesperidoside could upregulate the expression of NFATc1 and then promotes RANKL-induced osteoclastogenesis. Increased NFAT1 also activated the TRAP, CTSK, and VATPase-d2 gene promoters^21,25^, which was consistent with our qPCR results.

As RANKL also plays an important role in activating the downstream NF - κ B, p38, AKT and c-Jun N-terminal kinase (JNK) pathways^25,26^, we explored the effect of isorhamnetin 3-O-neohesperidoside on these osteoclast-related pathways downstream of RANKL. The phosphorylation levels of p38 and AKT but not P65 and JNK were enhanced by isorhamnetin 3-o-neohesperidin. In brief, isorhamnetin 3-O-neohesperidoside promoted RANKL-induced osteogenesis in a multitargeted manner, targeting the NFATc1, p38 and AKT pathways.

Tooth eruption can be divided into 5 stages: pre-eruptive movement, intra-osseous eruption, mucosal penetration, pre-occlusal eruption, and post-occlusal eruption^1^. The resorption of crown-covered bone is essential for the establishment of intraosseous eruption. Consistent with its pro-osteoclastogenic and pro-resorptive properties *in vitro*, isorhamnetin 3-O-neohesperidoside promoted osteoclast differentiation and crown-covered bone resorption *in vivo*. More TRAP-positive osteoclasts formed in the isorhamnetin 3-o-neohesperidin-treated groups. The resorption of crown-covered bone was faster in the isorhamnetin 3-o-neohesperidin-treated groups than that in the control groups.

Interestingly, western blotting showed that isorhamnetin 3-O-neohesperidoside upregulated RANKL protein expression in crown coverage of dental follicle. Osteoclastogenesis in the coronal alveolar bone, which is essential to create an eruption pathway, was reported to be mediated by RANKL signaling^2,27,28^. Mouse tooth germ development is suppressed by exogenous osteoprotegerin (OPG), an inhibitor of RANK-RANKL signalling that acts as a decoy receptor of RANKL. RANKL-deficient mice developed severe osteopetrosis as well as tooth eruption defects^29^. Immunofluorescence showed positive RANKL expressions in the crown coverage of dental follicles. *In vitro* results confirmed that Isorhamnetin 3-O-neohesperidoside could promoted RANKL-induced osteogenesis by the NFATc1, p38 and AKT pathway. Increased RANKL in the coronal dental follicle induced by isorhamnetin 3-o-neohesperid further promoted crown-covered bone resorption *in vivo*. The dental follicle is essential for tooth eruption^30,31^. Disturbance in the functions of dental follicles results in delayed tooth eruption in cleidocranial dysplasia (CCD) patients^32,33^. However, the regulatory mechanisms of dental follicles in tooth eruption are still unclear^34,35^. RANKL can be secreted by osteocytes^36,37^ and dental follicle cells^38,39^. To elaborate the mechanisms by which RANKL expression in the coronal dental follicle is increased by isorhamnetin 3-O-neohesperid, RANKL-related signalling pathways and transcriptional factors in dental follicle cells and osteocytes are worthy of further study in future.

Taken together, these results demonstrate isorhamnetin 3-O-neohesperidoside promoted the RANKL-induced osteogenesis of BMMs by NFATc1, p38 and AKT pathways *in vitro* and aggravated crown-covered bone resorption *in vivo*. If tooth eruption delayed, active treatment is recommended^40^. Isorhamnetin 3-O-neohesperidoside may be a candidate therapeutic for the treatment of delayed intraosseous eruption.

## Methods

### Animals

Six C57BL/6 mice (Postnatal day 7) with weights ranging from 3.2–6.4 g (average of 4.5 g) were chosen and cared for according to the Guidelines for Ethical Conduct in the Care and Use of Animals. All experimental protocols in this study were carried out in accordance with relevant guidelines and regulations and approved by the Ethics Committee of Shanghai Ninth People′s Hospital, Shanghai Jiao Tong University School of Medicine (SH9H-2019-A502–1). To observe crown-covered bone resorption during development of the osseous eruption canal, the right mandibular first molar received the local administration of 18.75 mg/kg isorhamnetin 3-O-neohesperidoside by gingival injection for 4 days, while the left mandibular first molar received saline as a control. The bilateral mandibles were collected at postnatal day 11 and 13 and then fixed in 4% paraformaldehyde for 24 h. After demineralization in 10% EDTA for 1 month, serial sections 5 mm in thickness were prepared in the mesial distal direction for TRAP staining as reported previously^2,41^.

### Cell culture

Bone marrow‐derived macrophages were isolated from the femurs and tibias of 6‐week‐old male C57BL/6 mice and cultured in α‐MEM with 10% FBS and 30 ng/ml M‐CSF in a humidified environment of 5% CO_2_ at 37 °C as reported previously^42^.

### Cell viability assay

BMMs were seeded into 96-well plates (8 × 10^3^ cells/well) in triplicate, and cultured in complete α‐MEM (10% FBS and 30 ng/ml M‐CSF) with isorhamnetin 3-O-neohesperidoside at a concentration (0.5, 1, 5, 10, 25, 50, 100 and 200 μM) for 24, 72, and 96 hrs. Ten microliters of CCK-8 solution was added to each well for 4 h, following which cell viability was determined by measuring the absorbance at 450 nm, as reported previously^16^.

### Osteoclast differentiation and TRAP staining

As reported previously^16^, BMMs were seeded into 96-well plates (1 × 10^4^ cells/well). After 24 h, the cells were cultured in α‐MEM (10% FBS, 30 ng/ml M‐CSF and 50 ng/mL RANKL) with isorhamnetin 3-O-neohesperidoside at a concentration gradient (0, 1, 5, 25 and 50 μM). The medium was changed every 2 days. After fixation with 4% paraformaldehyde, TRAP staining solution was applied to the cells. TRAP-positive cells with more than three nuclei were counted as osteoclasts, which were analysed using Image J software.

### Bone resorption assay

Corning Osteo Assay plates (Corning, NY, USA) with a bone biomimetic synthetic surface were used. BMMs (2 × 10^4^ cells/well) were cultured in complete α‐MEM (10% FBS, 30 ng/ml M‐CSF and 50 ng/mL RANKL) with isorhamnetin 3-O-neohesperidoside at a concentration gradient (0, 1, 5, 25 and 50 μM) for 9 days. The osteoclasts were then removed by incubation with 5% sodium hypochlorite for 5 min. The total resorption area was analysed using Image J software^25,42^.

Bovine bone slices in 96‐well plates were used for an improved bone resorption assay. BMMs (2 × 10^4^ cells/well) were cultured in complete α‐MEM (10% FBS, 30 ng/ml M‐CSF and 50 ng/mL RANKL) with isorhamnetin 3-O-neohesperidoside at two concentrations (0 and 50 μM) for 9 days. The OCs were then removed by incubation with 5% sodium hypochlorite for 5 min. Resorption was visualized under a scanning electron microscope at 5.0 kV. Five viewing fields from each bone slice were randomly selected for further analysis. Resorption areas were quantified using ImageJ software, as reported previously^43^.

### Quantitative PCR analysis

Quantitative PCR was conducted as previously described^25,42^. Total RNA was obtained using TRIzol reagent (Takara Biotechnology, Shiga, Japan). A PrimeScript RT Reagent Kit (TaKaRa Biotechnology) was then used to obtain cDNA. A TB Green Premix Ex TaqTM Kit (TaKaRa Biotechnology) was applied for qPCR. The following primers were used to detected osteoclastogenic genes used in this study: mouse NFATc1: forward, 5′-TGCTCCTCCTCCTGCTG CTC-3′ and reverse, 5′-GCAGAAGGTGGAGGTGCAGC-3′; mouse CTSK: forward, 5′-CTTCCAATACGTGCAGCAGA-3′ and reverse, 5′-TCTTCAGGGCTTTCTCGTTC-3′; mouse VATPase d2: forward, 5′-AAGCCTTTGTTTGACGCTGT-3′ and reverse 5′-TTCGATGCCTCTGTGAGATG-3′; mouse TRAP: forward, 5′-CTTCCAATACGTGCAGCAGA-3′ and reverse, 5′-CCCCAGAGACATGATGAAG TCA-3′; and mouse GAPDH: forward, 5′-CACCATGGGAGAAGGCCGGGG-3′ and reverse, 5′-GACGGACACATTGGGGGTAG-3′.

### Western blotting

Western blotting was carried as previously described^25,42^. The samples were incubated in sodium dodecyl sulfate (SDS) lysis buffer (Beyotime, China) supplemented with protease inhibitor cocktail (Beyotime). Proteins were separated by 10% SDS‐polyacrylamide gel electrophoresis (PAGE) and then transferred to polyvinylidene difluoride membranes. After blocking in 5% (w/v) skim milk for 1 h, the membranes were incubated with the primary antibodies (anti-β-actin, 1:1000; (anti-p-AKT, 1:1000; anti-AKT, 1:1000; anti-p-p38, 1:1000; anti-p38, 1:1000; anti-p-p65, 1:1000; anti-p65, 1:1000; anti-p-JNK, 1:1000; anti-JNK, 1:1000; anti-NFATc1 1:1000, anti-RANKL 1:1000 and anti-GAPDH) overnight at 4 °C, and then incubated with appropriate secondary antibodies for 1 h at room temperature. Odyssey Infrared Imaging System (Li‐COR Biosciences, Lincoln, NE) was used for exposing blots.

### Immunofluorescence

Immunofluorescence was performed as previously described^16,18^. Polyclonal antibody against RANKL (dilution 1:500, Abcam, UK) was applied. The sections were incubated with rhodamine (TRI-TC)-conjugated goat anti-rabbit IgG (Sigma, USA) for 1 h at room temperature. Nuclei were stained with a DAPI solution (Sigma, USA) for 5 min. PBS was used as a control.

### Statistical analysis

All data are expressed as the mean ± standard deviation. Student’s t‐tests, one-way analysis of variance and the Newman–Keuls test were conducted with GraphPad Prism 5 software. Differences with a p‐value of less than 0.05 were considered to be statistically significant.

## Supplementary information

Supplementary information.
