# Supplementary material for: Isorhamnetin 3-O-neohesperidoside promotes the resorption of crown-covered bone during tooth eruption by osteoclastogenesis
Source: Sci Rep. 2020 Mar 20;10:5172. doi: 10.1038/s41598-020-62107-7 (PMC7083939; doi:10.1038/s41598-020-62107-7)
Supplement: Supplementary file 1 — Supplementary information. [file 41598_2020_62107_MOESM1_ESM.pdf]

Isorhamnetin 3-O-neohesperidoside promotes the resorption of crown-covered bone during tooth eruption by osteoclastogenesis

Xijiao Yu[1,2#], Fuju Zheng[2#], Wenzhi Shang[2#], Yanmei Du[2], Jinze Zhen[1*], Yi Mao[1*], Shanyong Zhang[1*]

1.Department of Oral Surgery, Ninth People's Hospital, College of Stomatology, Shanghai Jiao Tong University School of Medicine, Shanghai Key Laboratory of Stomatology & Shanghai Research Institute of Stomatology, Shanghai, People's Republic of China
2. Department of Endodontics, Jinan Stomatological Hospital, Jinan, Shandong 250001.
3. Department of Oral Maxillofacial Head Neck Oncology, Ninth People's Hospital, College of Stomatology, Shanghai Jiao Tong University School of Medicine, Shanghai Key Laboratory of Stomatology & Shanghai Research Institute of Stomatology, Shanghai, People's Republic of China.
# These authors contributed equally: Xijiao Yu, Fuju Zheng and Wenzhi Shang
*Correspondence to: Dr Shangyong Zhang, Mingyi Wang and Yi Mao.
Shangyong Zhang and Yi Mao Department of Oral Surgery, Ninth People's Hospital, College of Stomatology, Shanghai Jiao Tong University School of Medicine, Shanghai Key Laboratory of Stomatology & Shanghai Research Institute of Stomatology, Shanghai, People's Republic of China.
E-mail: zhangshanyong@126.com; maoyi1994@126.com;zhenlich@163.com.

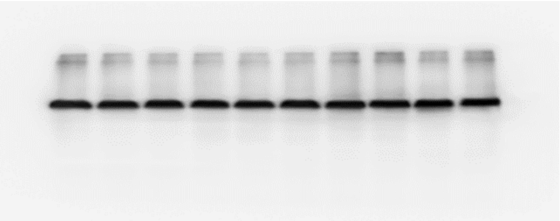

AKT

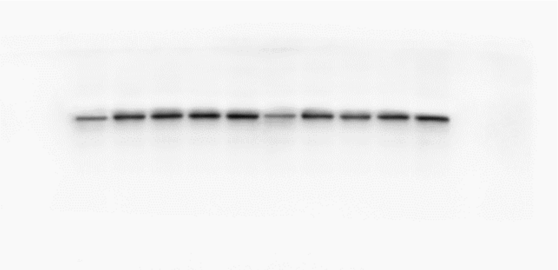

p AKT

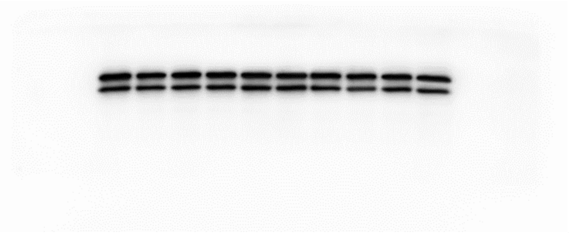

JNK

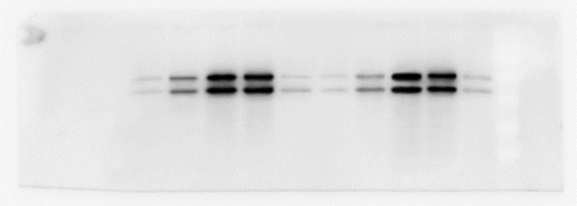

P JNK

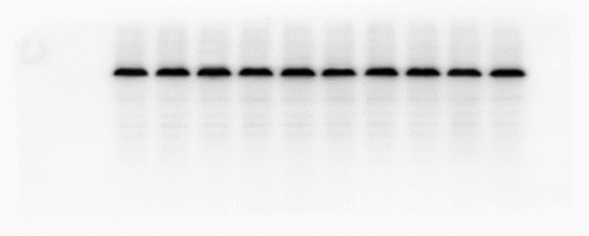

P65

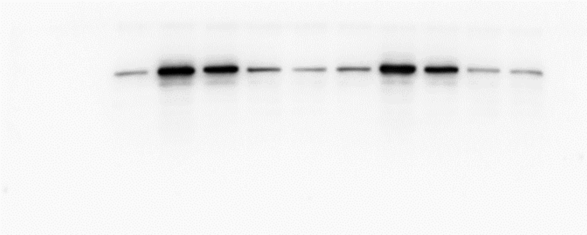

P P65

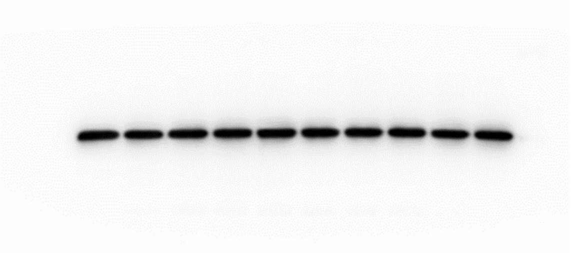

p38

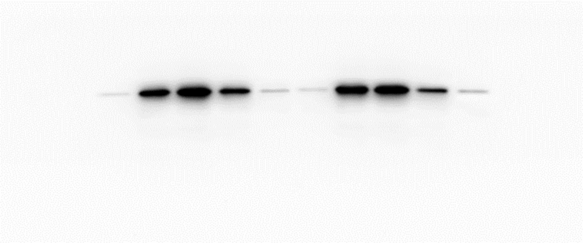

p p38

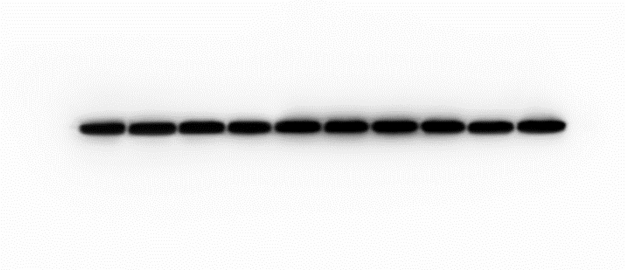

B-action

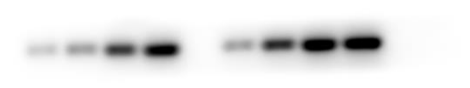

NFATc1

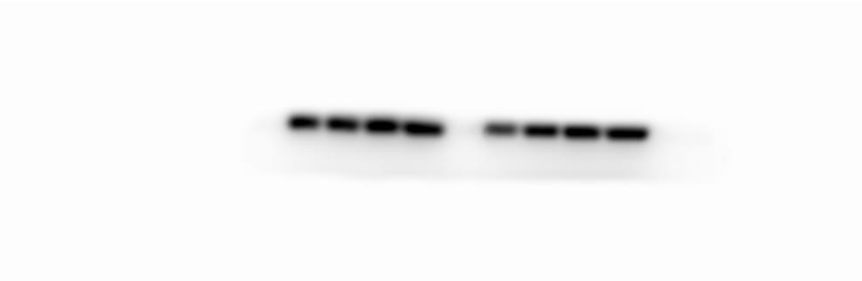

B-action

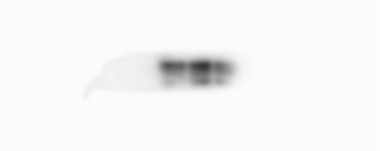

RANKL

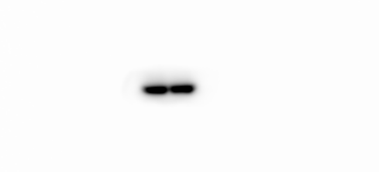

GAPDH
